# Genetic diversity and population structure of *Passiflora* spp. using inter-primer binding site (iPBS) – retrotransposon markers

**DOI:** 10.1007/s11033-026-12038-9

**Published:** 2026-06-01

**Authors:** Onildo Nunes de Jesus, Eva Maria Rodrigues Costa, Fernanda Quintanilha Azevedo, Vandeson Rodrigues de Sousa, Zanon Santana Gonçalves, Lucas Kennedy Silva Lima, Taliane Leila Soares

**Affiliations:** 1 Embrapa Mandioca e Fruticultura, Rua Embrapa, s/n, Cruz das Almas, P.O. Box 007, Chapadinha, Bahia, 44380-000 Brazil; 2 Universidade Estadual de Feira de Santana, Av. Transnordestina, s/n, Novo Horizonte, Feira de Santana, Bahia, 44036-900 Brazil; 3 Embrapa Clima Temperado, BR 392, km 78 - 9º Distrito, Monte Bonito, P.O. Box 403, Pelotas, 96010-971 Rio Grande do Sul Brazil

**Keywords:** Passiflora, Genetic relationship, iPBS markers, Genetic resources

## Abstract

**Background:**

The *Passiflora* genus presents a representative variability of passion fruit species with food, pharmacological and ornamental potential. To understand this genetic variability, it is essential that genetic resources are well characterized for future use in breeding and conservation programs. The aim of this study was to investigate for the first time the diversity genetic and structure population of 88 *Passiflora* genotypes with the use of inter-primer binding site (iPBS) markers.

**Methods and results:**

Genotypes were grouped through Unweighted Pair-Group Mean Average (UPGMA). The population structure analysis was carried out by two methods using model-based Structure analysis and cluster-based discriminant analysis of principal components (DAPC). Samples of 88 genotypes were analyzed using 24 iPBS markers, of which 19 of them generated 234 informative bands, with an average of 12.32 per locus, ranging from 5 to 18 alleles. Polymorphic information content (PIC) values varied between 0.14 and 0.43, with an average value of 0.28. Nei’s genetic diversity (*He*) as 0.28 and Shannon’s information index (*I*) as 0.43. The genetic dissimarity for the 88 *Passiflora* genotypes ranged from 0.11 to 0.97, with mean values of 0.74, indicating substantial genetic diversity. Molecular variance analysis (AMOVA) indicated that within group variations contributed more (51%) to the genetic diversity than among group variations (49%), with percetage of the genetic differentiation coefficient (*F*_*ST*_= 0.49). Discriminant analysis of principal components (DAPC) corroborated by cluster analysis (UPGMA) and Bayesian model-based analysis of population genetic structure categorized all materials into ten groups. Notably, a substantial portion of genotypes clustered within populations correlated with the *Passiflora* species. The ornamental hybrids were all discriminated based on the iPBS markers used and the G9 group allowed for the allocation of at least six of them.

**Conclusions:**

The results of this study provide valuable information for passion fruit germplasm management and contribute to the improvement of current breeding strategies. They also demonstrated the effectiveness of iPBS retrotransposon markers in detecting genetic differentiation and reliably elucidating genetic relationships among passion fruit genotypes, thereby supporting breeding programs and germplasm conservation efforts.

**Supplementary Information:**

The online version contains supplementary material available at 10.1007/s11033-026-12038-9.

## Introduction

*Passiflora* is the richest genus of the Passifloraceae family, comprising more than 500 species (of which at least 50 are edible) [1, 2]. Most of its species are widely cultivated in tropical and subtropical regions worldwide because of their multifaceted benefits, including food, ornamental and medicinal purposes [3–5] due to its contents of alkaloids, flavonoids and carotenoids, minerals and vitamins A, C, and D [6].

Brazil ranks as the largest producer and consumer of passion fruit in the world, reaching production in 2024 of more than 736 thousand tons, in a harvested area with around 47 thousand hectares [7], standing out in the fruit agribusiness. In Brazilian plantations, *Passiflora edulis* Sims (sour passion fruit) is the most representative species, with approximately 95% of the planted area, followed by *P. alata* (sweet passion fruit).

The Embrapa Mandioca e Fruticultura germplasm bank contains 490 genotypes, including commercial and wild species of *Passiflora*, of which only a third has been explored, which indicating the importance of conserving these genetic resources. The wide genetic variability of this genus makes it a valuable source of genes for tolerance to the main biotic and abiotic stressors affecting passion fruit. Furthermore, much of the potential for use in breeding programs and pharmacological applications of species in this genus has not yet been evaluated, making it important to conserve these genetic resources.

Germplasm characterization is fundamental for understanding its genetic variability [6], to support germplasm conservation, and identify the pharmacological potential [8]. Traditionally, the genetic diversity of passion fruit is estimated through morphological characters, including those related to stems, leaves, flowers, fruits and seeds [9, 10], as well as agronomic traits such as fruit quality, yield, and disease resistance [11–13]. However, these traits are influenced by environmental factors and developmental stages. This can limit their accuracy in reflecting genetic relationships, so that the results are not always consistent with the variability estimated based on molecular markers [14]. In contrast, the molecular markers provide direct estimation of genetic variation at the DNA level and are not influenced by environmental factors, hence being one of the most precise and efficient methods to analyze species’ genetic diversity [15, 16].

Several molecular marker techniques have been employed to analyze genetic diversity among different *Passiflora* species. Among them, we can mention amplified fragment length polymorphism – AFLP [10], inter-simple-sequence repeat -ISSR [17], simple-sequence repeat – SSR [2], random amplified polymorphic DNA – RAPD [18], resistance gene analog – RGA [19], single-nucleotide polymorphism – SNP [20], and next-generation sequencing – NGS [21]. Each of these molecular marker methods offers distinct advantages and contributes to a comprehensive understanding of the genetic variability of passion fruit.

Although several molecular tools are available for assessing and revealing genetic diversity in plant breeding, retrotransposons are genetic elements that constitute major components of most eukaryotic genomes, representing approximately 50–90% of plant genomes [22]. As reported for other species, partial genome sequencing of *Passiflora* has demonstrated a high abundance of retrotransposon-rich regions [23], highlighting their potential use as molecular markers. Among the markers derived from these regions, inter-primer binding site (iPBS) markers, classified as dominant markers, have become increasingly preferred in recent years for genetic diversity studies due to their universal applicability. These markers exhibit high values for total amplified fragments, polymorphic amplified fragments, polymorphism information content, marker index, and resolving power, in addition to detecting higher levels of genetic variation compared with other molecular marker systems [24, 25]. Furthermore, iPBS retrotransposon markers offer several additional advantages, including technical simplicity, high informativeness, low cost, no requirement for prior genomic information, high reproducibility, and low environmental influence [15, 26–28]. These markers have been successfully applied to evaluate genetic diversity, population structure, and evolutionary relationships in numerous plant species [15, 16, 29–31]. To the best of our knowledge, no previous studies have characterized passion fruit germplasm using iPBS retrotransposon markers.

Despite the economic importance and different potential uses of passion fruit, there is only a limited understanding on germplasm diversity of this species. Therefore, the present study explored for the first time the genetic variability of 88 *Passiflora* genotypes using iPBS-retrotransposon markers, with the aim of elucidating the parental relationships and population genetic structures of these genetic resources. The findings of this work provide a solid foundation for future use in conservation and breeding programs.

## Materials and methods

### Plant material and experimental locale

The study included 88 genotypes belonging to different *Passiflora* species (Table 1). These genotypes were obtained from the Passion Fruit Germplasm Bank of Embrapa Mandioca e Fruticultura located in the municipality of Cruz das Almas, Bahia, Brazil (12° 39’ 25” S, 39° 07’ 27” W, 226 m). Fresh young leaf samples from three individual plants were harvested, immediately frozen on dry ice, and transported for storage in an ultralow temperature freezer at − 80 °C for subsequent DNA extraction.

**Table 1:** Passion fruit genotypes used in molecular characterization with iPBS retrotransposons

Number	Genotype	Code^1^	Scientific nome	Country	State
1	BGP004	*Pal*	*Passiflora alata* Curtis	Brazil	SP
2	BGP008	*Pgi*	*Passiflora gibertii* N.E.Br.	Brazil	BA
3	BGP016	*Pci*	*Passiflora cincinnata* Mast.	Brazil	AL
4	BGP018	*Ped*	*Passiflora edulis* Sims	Brazil	MG
5	BGP032	*Ped*	*Passiflora edulis* Sims	Brazil	PR
6	BGP038	*Ped*	*Passiflora edulis* Sims	Brazil	PA
7	BGP043	*Ped*	*Passiflora edulis* Sims	Brazil	-
8	BGP046	*Pedm*	*Passiflora edmundoi* Sacco	Brazil	MT
9	BGP049	*Ped*	*Passiflora edulis* Sims	Brazil	MT
10	BGP074	*Pga*	*Passiflora galbana* Mast.	Brazil	BA
11	BGP077	*Pci*	*Passiflora cincinnata* Mast.	Brazil	BA
12	BGP105	*Pte*	*Passiflora tenuifila* Killip	Brazil	SP
13	BGP107	*Pmo*	*Passiflora morifolia* Mast.	Brazil	SP
14	BGP109	*Pga*	*Passiflora galbana* Mast.	Brazil	-
15	BGP112	*Psu*	*Passiflora suberosa* L.	Brazil	-
16	BGP114	*Pmu*	*Passiflora muchronata* Lam.	Brazil	SP
17	BGP125	*Pru*	*Passiflora rubra * *L.*	Brazil	SP
18	BGP134	*Psu*	*Passiflora suberosa* L.	Brazil	BA
19	BGP137	*Pga*	*Passiflora galbana* Mast.	Brazil	BA
20	BGP143	*Psu*	*Passiflora suberosa* L.	Brazil	SP
21	BGP152	*Psu*	*Passiflora suberosa* L.	Brazil	SP
22	BGP153	*Pfo*	*Passiflora foetida* L.	Brazil	SP
23	BGP157	*Pqu*	*Passiflora quadrangulares* L.	Brazil	BA
24	BGP162	*Pal*	*Passiflora alata* Curtis	Brazil	BA
25	BGP164	*Ped*	*Passiflora edulis* Sims	Brazil	BA
26	BGP165	*Ped*	*Passiflora edulis* Sims	Brazil	BA
27	BGP170	*Pmal*	*Passiflora mallacophyla* Mast.	Brazil	-
28	BGP172	*Pra*	*Passiflora racemosa* Brot.	Brazil	-
29	BGP180	*Ped*	*Passiflora edulis* Sims	Brazil	SP
30	BGP185	*Ped*	*Passiflora edulis* Sims	Brazil	SP
31	BGP188	*Ped*	*Passiflora edulis* Sims	Brazil	SP
32	BGP193	*Psu*	*Passiflora suberosa* L.	Brazil	-
33	BGP198	*Pgi*	*Passiflora gibertii* N.E.Br.	Brazil	-
34	BGP205	*Ped*	*Passiflora edulis* Sims	Brazil	SP
35	BGP222	*Ped*	*Passiflora edulis* Sims	Brazil	BA
36	BGP223	*Ped*	*Passiflora edulis* Sims	Brazil	BA
37	BGP224	*Ped*	*Passiflora edulis* Sims	Brazil	BA
38	BGP232	*Pal*	*Passiflora alata* Curtis	Brazil	SP
39	BGP237	*Pse*	*Passiflora setacea* DC.	Brazil	BA
40	BGP238	*Pse*	*Passiflora setacea* DC.	Brazil	BA
41	BGP242	*Pse*	*Passiflora setacea* DC.	Brazil	BA
42	BGP268	*Pci*	*Passiflora cincinnata* Mast.	Brazil	BA
43	BGP277	*Ped*	*Passiflora edulis* Sims	Brazil	BA
44	BGP297	*Pci*	*Passiflora cincinnata* Mast.	Brazil	BA
45	BGP298	*Pci*	*Passiflora cincinnata* Mast.	Brazil	BA
46	BGP318	*Pmo*	*Passiflora morifolia* Mast.	Brazil	SP
47	BGP322	*Pci*	*Passiflora cincinnata* Mast.	Brazil	BA
48	BGP324	*Ped*	*Passiflora edulis * *Sims*	Brazil	DF
49	BGP328 (BRS-GA)	*Ped*	*Passiflora edulis* Sims	Brazil	DF
50	BGP330	*Ped*	*Passiflora edulis* Sims	Brazil	BA
51	BGP334	*Ped*	*Passiflora edulis* Sims	Brazil	BA
52	BGP340	*Ped*	*Passiflora edulis* Sims	Brazil	BA
53	BGP343	*Ped*	*Passiflora edulis* Sims	Brazil	BA
54	BGP346	*Pci*	*Passiflora cincinnata* Mast.	Brazil	BA
55	BGP391	*Ped*	*Passiflora edulis* Sims	Brazil	BA
56	BGP392	*Ped*	*Passiflora edulis* f. *edulis* Sims	Brazil	RS
57	BGP393	*Pal*	*Passiflora alata* Curtis	Brazil	RJ
58	BGP394	*Psub*	*Passiflora subrotunda* Mast.	Brazil	CE
59	BGP395	*Pfo*	*Passiflora foetida* L.	Brazil	BA
60	BGP398	*Pci*	*Passiflora cincinnata* Mast.	Brazil	BA
61	BGP400	*Ped*	*Passiflora edulis* Sims	Brazil	BA
62	BGP401	*Ped*	*Passiflora edulis* Sims	Brazil	BA
63	BGP405	*Pse*	*Passiflora setacea* DC.	Brazil	BA
64	BGP406	*Pci*	*Passiflora cincinnata* Mast.	Brazil	BA
65	BGP408	*Pco*	*Passiflora coccinea* Aubl.	Brazil	SP
66	BGP413	*Pci*	*Passiflora cincinnata* Mast.	Brazil	SP
67	BGP414	*Pgi*	*Passiflora gibertii* N.E.Br.	Brazil	BA
68	BGP417	*Pba*	*Passiflora bahiensis* Klotzsch	Brazil	BA
69	BGP436	*Ped*	*Passiflora edulis* Sims	Brazil	BA
70	BGP436a	*Ped*	*Passiflora edulis* Sims	Brazil	-
71	BGP436b	*Ped*	*Passiflora edulis* Sims	Brazil	-
72	BGP437	*Pse*	*Passiflora setacea* DC.	Brazil	BA
73	BRS-MC	*Pal*	*Passiflora alata* Curtis	Brazil	-
74	BRS-PC	*Pse*	*Passiflora setacea* DC.	Brazil	-
75	BGP172 X BGP008	Orn	*P. racemosa x P. gibertii*	Brazil	BA
76	BGP172 x BGP038	Orn	*P. racemosa x P. edulis*	Brazil	BA
77	BGP172 x BGP114	Orn	*P. racemosa x P. mucronata*	Brazil	BA
78	BGP172 X BGP322	Orn	*P. racemosa x P. cincinnata*	Brazil	BA
79	BGP008 x BGP330	Orn	*P. gibertii x P. edulis*	Brazil	BA
80	BGP074 x BGP046	Orn	*P. galbana x P. edmundoi*	Brazil	BA
81	BGP074 x BGP085	Orn	*P. galbana x P. gibertii*	Brazil	BA
82	BGP074 x BGP172	Orn	*P. galbana x P. racemosa*	Brazil	BA
83	BC2.1	In.h	*RC1-H44 x BGP224*	Brazil	BA
84	BC2.18	In.h	RC1-H44 x BGP224	Brazil	BA
85	BC2.2	In.h	RC1-H44 x BGP225	Brazil	BA
86	BC2.4	In.h	RC1-H57 x BGP224	Brazil	BA
87	BC2.5	In.h	RC1-H57 x BGP223	Brazil	BA
88	BC2.8	In.h	RC1-H44 x BGP223	Brazil	BA

### DNA extraction

The DNA extraction process from the 88 genotypes involved the preparation of DNA from fresh young leaves, which were submitted to extraction at the Laboratory of Molecular Biology of Embrapa Mandioca e Fruticultura. Genomic DNA extraction followed the standard CTAB technique described by [32], with some modifications. The DNA quantification was performed after electrophoresis of aliquots of each sample, comparing them with a series of known concentrations of DNA Lambda (Invitrogen) in 1.0% agarose gels (w/v). The fragments were visualized by staining with ethidium bromide (1.0 µg mL^− 1^). Each DNA sample was diluted to a final concentration of 5 ng µL^− 1^ for iPBS analysis.

### PBS-retrotransposon amplification

In this experiment, 24 iPBS primers were initially tested, from which 19 primers exhibiting high levels of polymorphism were selected for subsequent analysis. The primers utilized were originally developed by [24]. The sequences are detailed in Table 1.

PCR involved the use of a 10X PCR buffer (500mM KCl, 100mM Tris-HCl pH 8,5), 0.6 µM primer, 0.2 mM of each dNTP, 2 mM MgCl_2_, 1 U Taq polymerase, and 10 µg/ng DNA template in a 15 µL reaction mixture. Amplification of iPBS primers was performed with a 95 °C denaturation cycle for 3 min followed by 30 cycles of 15 s at 95 C, 60 s at 68 °C, and 60 s at 72 °C, concluding with a final extension step of five minutes at 72 °C, followed by 4 °C until the sample was removed from the thermocycler. The amplification products were separated by electrophoresis (80 V for 7 h) in a 2.5% agarose gel. The gels were stained with ethidium bromide (0.5 µg/mL) and visualized under UV light. The amplicons were compared with a 100 bp DNA ladder.

### Genetic diversity of *Passiflora*

Genetic diversity analysis of *Passiflora* genotypes was performed using 19 iPBS markers (grouping 234 distinct alleles). To this end, 88 *Passiflora* genotypes belonging to 10 natural populations were evaluated. The original data were scored in binary form (0 or 1), and the results were processed using Excel. A value of 0 denotes the absence of a band, while a value of 1 represents the presence of a band. Missing values (-9) were treated by imputation.

Initially, the potential of iPBS markers to estimate genetic variability of *Passiflora* spp. was examined by calculating the marker informativeness, namely total number of amplified bands (TNB); number of shared bands (NSB), Nei’s genetic diversity index (*He*), Shannon’s index (*I*), and polymorphic information content (PIC), as observed and estimated for each primer. The classification of the informativeness of dominant markers based on PIC values followed the classification proposed by [33] with some modifications: low (0 to 0.10), medium (0.11 to 0.25), high (0.26 to 0.40); and very high (0.41 to 0.50).

A dissimilarity matrix was constructed using the Jaccard similarity index and clustering was conducted using the unweighted pair group method with arithmetic mean (UPGMA) method. The cophenetic correlation coefficient was determined by comparing the Jaccard similarity matrix with its corresponding dendrogram.

The genetic structure of the genotypes was analyzed through a Bayesian approach and discriminant analysis of principal components (DAPC). For the Bayesian approach, the Structure v.2.3.2 software used to define a value of K groups that best represented the 88 genotypes. This analysis incorporated Markov chain Monte Carlo (MCMC) simulations, executed with a burn-in period of 10,000 iterations followed by 100,000 iterations under a model permitting admixture and correlated allele frequencies. The choice of the probable population number was made based on the highest natural log-transformed value of the likelihood (*ln* X/K) of the data.

In addition to the Bayesian model, the population genetic structure was investigated using DAPC (Discriminant Analysis of Principal Components). This method combines principal component analysis and discriminant analysis. The cumulative variance of the PCAs was calculated to determine the amount of information retained. The xvalDapc cross-validation function was used to determine the correct number of PCAs to use and the number of discriminant functions to save for DAPC. The DAPC results were visualized using scatterplots of the discriminant functions, allowing the separation of the population groups to be observed. Additionally, the proportion of variation explained by each discriminant function (DA eigenvalues) was calculated, indicating the relative contribution of each discriminant axis to the separation of the groups. PCA and DAPC analyses were performed in the R software, using the Adegenet packages [34].

Additionally, the genetic structure between and within the group was assessed using analysis of molecular variance (AMOVA). Partitioning of total genetic variation within and among gene pool identities was also determined. The significance of the variance components was tested by random permutation (*n* = 999), obtaining p-values associated with each hierarchical level. The result was expressed in terms of the proportion of variance explained by each component (R²), allowing the quantification of the relative contribution of intergroup genetic differentiation and intragroup variation. The coefficient of genetic differentiation (F_ST_) was also estimated using groups previously defined by clustering analysis. Binary data processing was then analyzed using the Adonis2 package in the R software [35].

## Results

### iPBS-retrotransposon amplification

**Fig. 1:**
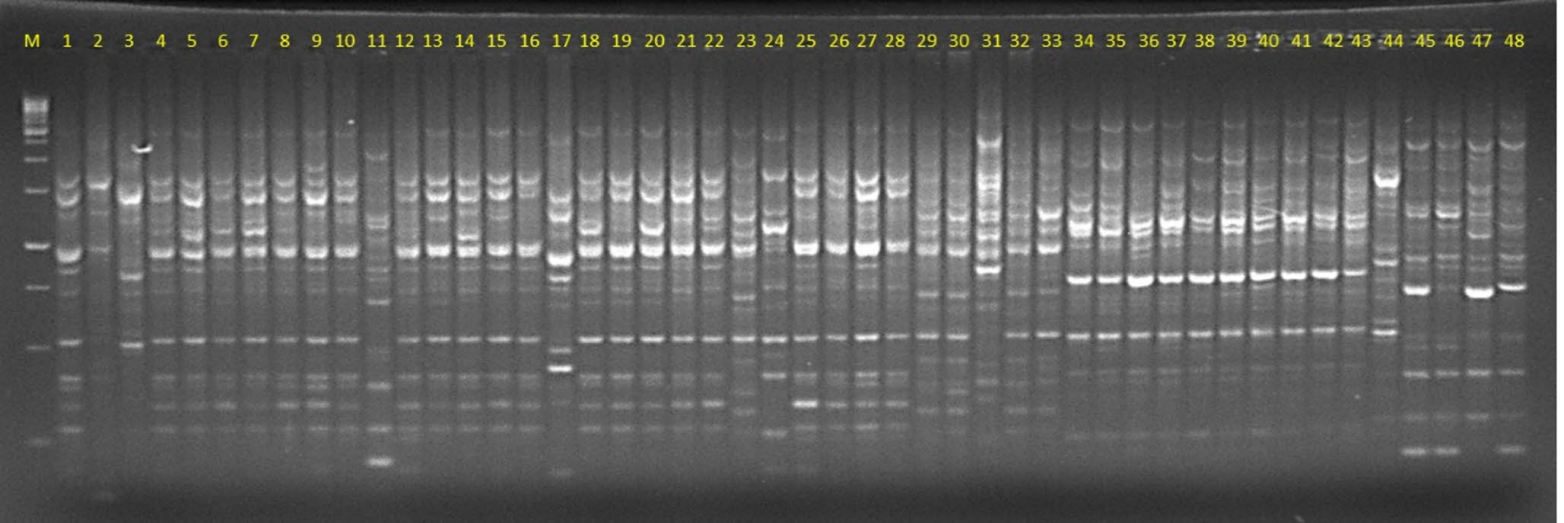
Agarose gel image of PCR products amplified with iPBS primer 2224. M = DNA ladder (100 bp); Codes 1–48 = PCR fragments from 48 genotypes listed in Table 1

We used the PCR-based iPBS marker technique to determine the genetic diversity of 88 *Passiflora* genotypes (Fig. 1). Among the 24 primer combinations tested, 19 (79.2%) successfully amplified DNA fragments, generating a total of 234 bands (Table 2). The total number of amplified bands per primer ranged from 5 (iPBS2277) to 18 (iPBS2224), with average of 12.31 bands per primer. Five primer combinations were not amplified (IPBS 2238, iPBS-2078, iPBS-2376, iPBS-2273, iPBS-2277). The number of bands shared by more than one genotype ranged from 7.88 for iPBS-2078 to 42.3 for iPBS-2079, resulting in an average of 19.79 alleles per locus.

**Table 2:** iPBS primers validated in 88 *Passiflora* genotypes. At °C: Annealing temperature, TNB: total number of bands produced by each primer, GNNA: genotype numbers that did not amplify (GNNA), number shared bands by more than one genotype (NSB), PIC: polymorphic information content, Nei’s genetic diversity (*He*) and Shannon’s information index (*I*)

Primers	AT °C	TNB	GNNA	NSB	PIC	*He*	*I*
iPBS 2074	49.6	15	0	14.33	0.33	0.33	0.49
iPBS 2221	55.6	9	5	20.23	0.23	0.23	0.36
iPBS 2224	55.4	18	1	22.40	0.32	0.32	0.48
iPBS 2232	55.4	16	13	16.33	0.31	0.31	0.47
iPBS 2237	55.0	17	9	23.22	0.20	0.20	0.33
iPBS 2239	55.0	13	2	11.59	0.31	0.31	0.47
iPBS 2373	51.0	12	4	16.60	0.24	0.24	0.38
iPBS 2075	52.0	7	2	33.57	0.40	0.40	0.59
iPBS 2076	59.2	12	13	10.50	0.14	0.14	0.25
iPBS 2077	55.1	11	7	31.09	0.37	0.37	0.54
iPBS 2079	65.2	10	5	42.30	0.43	0.43	0.62
iPBS 2095	55.0	12	10	12.83	0.17	0.17	0.28
iPBS 2272	55.0	10	4	21.00	0.22	0.22	0.36
iPBS 2375	54.0	14	13	12.50	0.25	0.25	0.40
iPBS 2377	54.0	14	0	20.40	0.31	0.31	0.47
iPBS 2386	51.0	9	10	13.07	0.28	0.28	0.44
iPBS 2389	51.0	8	10	15.00	0.31	0.31	0.47
iPBS 2393	51.0	13	6	23.07	0.27	0.27	0.43
iPBS 2394	56.5	14	4	16.11	0.24	0.24	0.38
iPBS 2238*	56.0	10	20	17.13	0.26	NC	NC
iPBS 2273*	56.5	11	26	18.15	0.36	NC	NC
iPBS 2277*	52.0	5	34	20.14	0.42	NC	NC
iPBS 2376*	52.0	10	19	21.27	0.24	NC	NC
iPBS 2078*	62.0	8	29	7.88	0.21	NC	NC
Average	54.52	12.32	6.21	19.79	0.28	0.28	0.43

^*^Primers not used in statistical analyses due to many amplification failures. NC: not computed

Genetic diversity indices revealed substantial variation among markers (Table 2). The polymorphism information content (PIC) values of the 19 primer pairs ranged from 0.14 (iPBS-2076) to 0.43 (iPBS-2079), with an average of 0.28 (Table 2). Ten primer pairs (41.67%) had medium PIC values, 12 (50%) had PIC values ranging from 0.26 to 0.40, while, only 2 primer pairs (8.33%) presented PIC values varying from 0.41 to 0.50, classified as having medium, high and very high informativeness, respectively. Among the 19 markers, Nei’s genetic diversity (*He*) values ranged from 0.14 (iPBS2076) to 0.43 (iPBS2079), with an average of 0.28, while Shannon’s information index (*I*) varied from 0.25 (iPBS2076) to 0.62 (iPBS2079), with a mean of 0.43.

### Cluster analysis of *Passiflora* germplasm resources

**Fig. 2:**
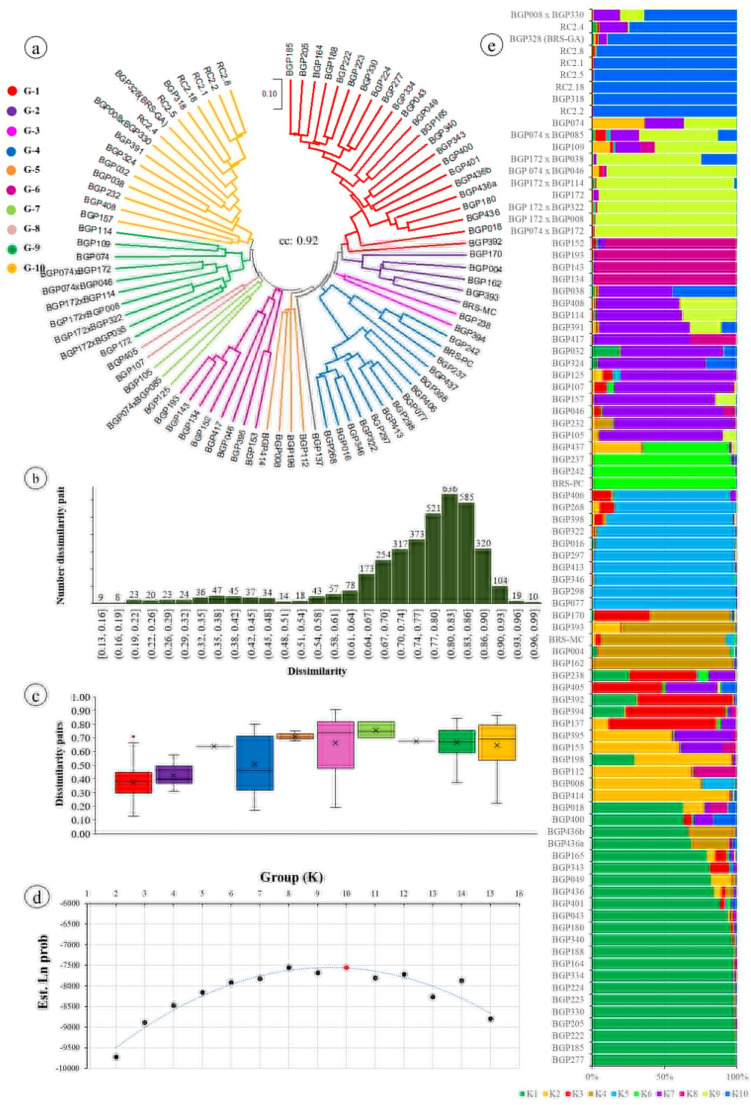
(**a**) Neighbor-joining dendrogram based on a Jaccard genetic dissimilarity matrix for 88 *Passiflora* spp. genotypes using 19 iPBS markers. (**b**) Distribution of genetic dissimilarity among genotype pairs. (**c**) Distribution of genetic dissimilarity among genotype pairs within each group (group colors correspond to those in the dendrogram). (**d**) Determination of the most likely number of subpopulations (K) among the evaluated accessions based on the mean lnP(X|K) values from 10 independent runs for each K. (**e**) Population structure of the genotypes inferred using a model-based clustering approach with admixture and correlated allele frequencies. The 10 groups are represented by different colors, and each vertical bar represents an individual genotype, partitioned into segments proportional to its estimated membership fraction in each subpopulation

**Fig. 3:**
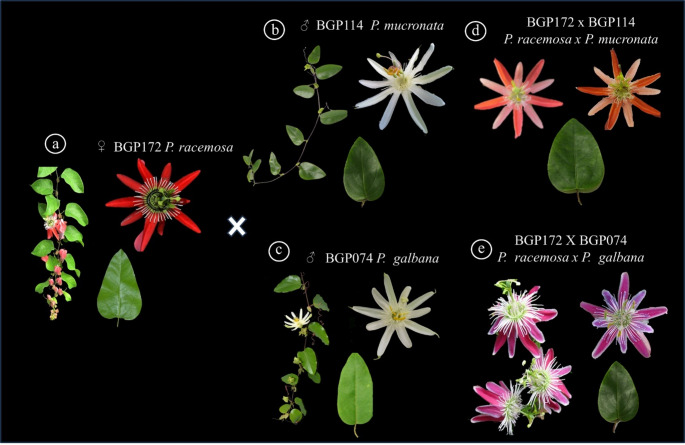
Aspect of the ornamental hybrids that were grouped in group G9. A-C) Parents. (**a**) BGP 172 - *P. racemosa*, (**b**) BGP 114 - P. *mucronata*, (**c**) BGP 074 - *P. galbana*. **d**-**e**) Hybrids formed. (**d**) BGP172 x BGP114 and (**e**) BGP172 x BGP074

Clustering analysis based on the Jaccard similarity coefficient and the unweight pair group method with arithmetic means (UPGMA) grouped the 88 *Passiflora* genotypes into ten distinct groups (Fig. 2a). The cophenetic correlation coefficient was *r* = 0.92. Group 1 (G1), consisting primarily of genotypes of 23 *P. edulis*. Group 2 (G2) was composed mainly of *P. alata* (4) and *P. mallacophya* (1). Group 3 (G3) was dominated by *P. setacea* (1) and *P. edulis* (1). Group 4 (G4) included *P. cincinnata* (10) and *P. setacea* (4). Group 5 (G5) was composed primarily of *P. suberosa* (1), and *P. gibertii* (3). Group 6 (G6) was composed exclusively of *P. suberosa* (4), *P. bahiensis* (1), *P. edmundoi* (1), *P. foetida* (2). Group 7 (G7) formed by *P. tenuifila* (1), *P. galbana* x *P. cincinnata* (1), *P. rubra* (1). Group 8 (G8) allocated *P. setacea* (1), *P. morifolia* (1). Group 9 (G9) represented by *P. racemosa* (1), *P. mucronata* (1), *P. galbana* (2) and six ornamental hybrids, including *P. racemosa* x *P. mucronata* (1), *P. racemosa* x *P. galbana* (1), *P. galbana* x *P. edmundoi* (1), *P. racemosa* x *P. cicinnata* (1), *P. racemosa* x *P. edulis* (1) and *P. racemosa* x *P. gibertii* (1), with morphological characteristics intermediate to their parents, such as variation in the size and color of the floral parts of the corona and also in the shape and size of the leaves (Fig. 3). Finally, Group 10 (G10) consisted of *P. edulis* (5), *P. morifolia* (1), *P. quadrangularis* (1), *P. coccinea* (1), *P. alata* (1), *P. gibertii* x *P. edulis* (1) and RC2 (6).

The dissimilarity among genotype pairs, calculated using the Jaccard index based on iPBS data from the 88 genotypes, ranged from 0.11 to 0.97, with an average of 0.74, indicating a wide range of genetic variability among the evaluated materials (Fig. 2b). The highest genetic dissimilarity (0.97) was observed between *P. cincinnata* genotypes (BGP016, BGP322, BGP413) and *P. edulis* (BGP391), while the lowest value (0.11) was found between *P. edulis* genotypes (BGP224 and BGP277). The highest frequency of genotype pairs occurred within the dissimilarity range of 0.67 to 0.90 (Fig. 2b). This amplitude was also corroborated by the low number of bands shared by more than one genotype, as given by the NSB values. (Table 2). The dissimilarity between the groups was heterogeneous (Fig. 2c), with greater amplitude observed in the G-4 and G-6 genotypes, with similarity values ranging from 0.17 to 0.80 and 0.19 to 0.90, respectively (Fig. 2c), probably related to the different species involved in the formation of this group.

The cophenetic correlation coefficient was determined by comparing the Jaccard similarity matrix with its corresponding dendrogram, obtained using the UPGMA clustering method. The cophenetic correlation coefficient was high (*r* = 0.92), indicating a good fit to the cluster analysis, so the dendrogram was a good representation of the iPBS data.

To investigate the population structure of the 88 *Passiflora* genotypes, we employed a model-based approach using the STRUCTURE software, which assigned each genotype to its specific subpopulations. The optimal value of K was determined based on the largest log-transformed likelihood value. The genetic structure analysis indicated that the optimal value of K was 10 (Fig. 2d), corresponding to 10 subgroups represented by the colors in the Fig. 2e.

Partitioning of total genetic variation within and between gene pool identities was performed using analysis of molecular variance (AMOVA), revealing that most of the variation (51%) was attributed to individuals within *Passiflora* populations, which indicates a high degree of variation among the individuals constituting these germoplasm (Table 3). Despite the lower importance of variation among in group (49%), the degree of intergroup genetic differentiation is very high (*F*_*ST*_ = 0.49) (Table 3).

**Table 3:** AMOVA results of iPBS data used to determine the genetic structure for different hierarchical levels of the 88 *Passiflora* genotypes

Source of variation	*DF	SQ	MSD	Variance (%)	F_ST_	*P*
Among group	9	13.008	0.1466	49%	0.49	0.001
Within group	78	12.102	0.1552	51%		
Total	87	25.110	0.3018	100%		

*DF: degrees of freedom, SQ: sum of squares, MSD: mean square deviation; variance; (%): Percentage of variance; *F*_ST_ (Φ_ST_): genetic differentiation coefficient (MSD_Among/MSD_Total). *P*: significance value

**Fig. 4:**
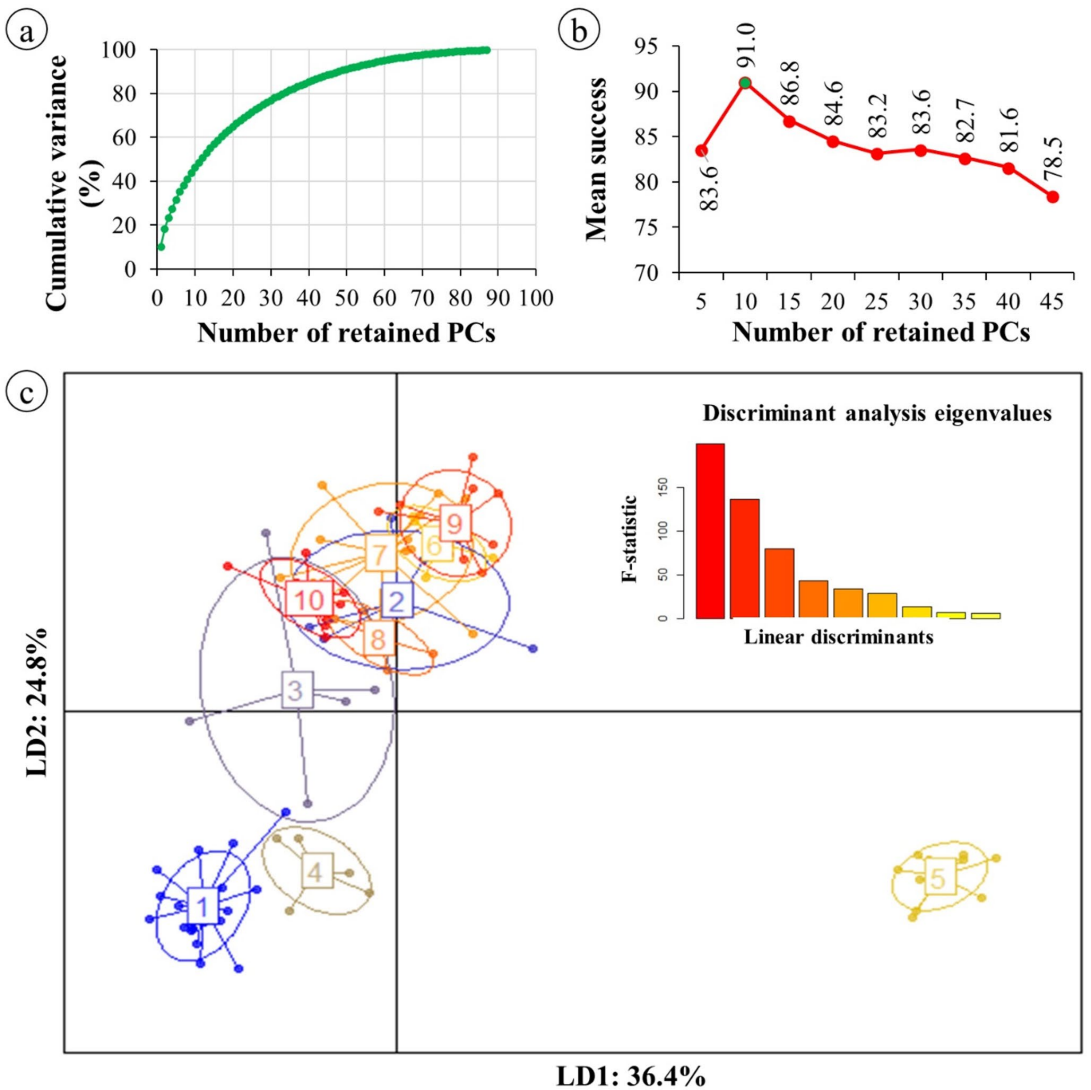
Discriminant analysis of principal components (DAPC) performed for 88 *Passiflora* genotypes. (**a**) Cumulative proportion of variance explained according to the number of retained principal components (PCs). (**b**) Cross-validation results showing the mean success of assignment as a function of the number of retained PCs; the optimal number of PCs retained is indicated by the highest assignment success. (**c**) Scatterplot of the first two linear discriminants (LD1 and LD2), explaining 36.4% and 24.8% of the among-group variation, respectively. Each dot represents an individual genotype and each ellipse represents a genetic cluster inferred by DAPC. Numbers indicate the identified subpopulations. The inset shows the eigenvalues of the discriminant functions

We initially conducted an exploratory multivariate analysis using principal coordinate analysis (PCoA) based on Jaccard genetic dissimilarity derived from 19 iPBS markers, which generated 234 distinct alleles across the 88 *Passiflora* genotypes. The first two coordinates collectively explained 28.55% of the total genetic variation, with PCoA1 accounting for 15.23% and PCoA2 for 13.32% (Supplementary Fig. 1). Although PCoA can be used as a clustering and visualization approach, the relatively low proportion of variation explained by the first two coordinates indicated a weakly structured pattern, characterized by broad genotype dispersion and the absence of clearly defined groups among the *Passiflora* genotypes (Supplementary Fig. 1). Therefore, Discriminant Analysis of Principal Components (DAPC) was subsequently employed to further investigate and corroborate genotype clustering according to their genetic backgrounds. DAPC proved effective in assigning *Passiflora* genotypes to specific groups. Based on cross-validation analysis, approximately 10 principal components (PCs) were retained during the preliminary data transformation step. Using these retained PCs, the first two discriminant functions explained 61.2% of the total between-group variation (Fig. 4a-b), revealing a clearer and more robust discrimination pattern among genotypes.

We performed DAPC to investigate the genetic structure of the dataset and to corroborate the number of genetic clusters inferred by the lnP(X|K) in Structure (K = 10). The cumulative variance curve indicated that the first principal component captured a large proportion of the total genetic variation, with diminishing gains as additional components were included (Fig. 4a). However, cross-validation analysis showed that using more than 10 principal components reduced classification accuracy, indicating overfitting (Fig. 4b). Therefore, we selected 10 PCs as the optimal number, balancing information retention and model generalization.

Based on these 10 PCs, the scatterplot of the first two discriminant functions clearly revealed the genetic structure of the population, showing well-defined groups among the 88 *Passiflora* genotypes (Fig. 4c). These two axes explained 61.2% of the total between-group variation, indicating that most of the genetic differentiation was well represented in the two-dimensional space. In addition, the high assignment success rate (91%) observed in the cross-validation procedure (Fig. 4b) further supports the reliability of the inferred group structure (Fig. 4c). The clustering pattern obtained by DAPC was consistent with the results observed in the UPGMA dendrogram (Fig. 2a) and the Bayesian model-based structure analysis (Fig. 2e), reinforcing the robustness of the inferred population structure and highlighting the effectiveness of iPBS markers for assessing genetic diversity of *Passiflora*.

## Discussion

Studies on genetic diversity are crucial for understanding the level of genetic variation and genetic structure within species [15]. Furthermore, it serves as a significant indicator for discerning and selecting superior genotypes, which can later be used in genetic improvement programs [16]. Over the years, many researchers have conducted extensive research on population structure and genetic diversity in different plant species [17, 20, 36, 37]. However, with advances in molecular biology, different approaches to population structure have shifted from phenotypic to molecular biology-based [36, 38].

Molecular markers represent an effective method for studying plant species genetic diversity, considering the limitations of morphological characterization, which are often influenced by the environment. In our study, we aimed to explore for the first time the genetic diversity and population structure in *Passiflor*a germplasm using iPBS-retrotransposon molecular markers.

Although no previous studies have employed iPBS markers specifically in *Passiflora*, these markers have been successfully used in other plant species, such as, *Pisum sativum* L [26], *Zanthoxylum* L [15], *Trigonella foenum-graecum* L [29], *Beta vulgaris* L [27], with 37 (44.6%), 10 (12.0%), 24 (28.9%) and 15 (18.0%) iPBS primers selected, respectively. In the present study it was possible to identify 19 (79.2%) of the 24 iPBS primers produced good polymorphic band profiles.

A set of 19 highly polymorphic iPBS-retrotransposon primers was employed to investigate the genetic variability and structure of populations of *Passiflora* species. The average number of bands per iPBS primers observed in this study (12.32 per primer) was higher when compared with data available for *P. edulis* using the dominant marker RAPD, with a mean of 6.8 bands per primer [39]. In contrast, other authors evaluating genetic diversity in *P. edulis* using different dominant markers observed the highest average number of bands when using ISSR markers with 28.2 [40] and 16.1 per primer [17], and also the use of SRAP with 133 bands per primer [41].

Nei’s gene diversity (*He*) and Shannon’s information index *(I*) were used to estimate the genetic variability among the evaluated genotypes. Higher expected heterozygosity (*He*) values indicate lower genetic uniformity and greater genetic diversity among genotypes. In the present study, *He* values among the 88 *Passiflora* genotypes ranged from 0.14 to 0.43, with an average value of 0.28. The iPBS2079 marker exhibited the highest *He* value (0.43), indicating a high level of genetic diversity among the evaluated *Passiflora* genotypes. Similarly, Shannon’s information index ranged from 0.25 to 0.62, with an average value of 0.43.

Previous studies assessing genetic diversity in *Passiflora* have reported variable results depending on the germplasm evaluated and, importantly, on the type of molecular marker employed [17]., analyzing five population of *P. edulis* with ISSR markers, reported mean *He* and *I* values of 0.18 and 0.27, respectively [41]. using sequence-related amplified (SRAP) investigate 22 *P. edulis* genotypes observed average values of 0.14 for *He* and 0.09 for *I* [40]., evaluating 21 genotypes of *Passiflora edulis* with ISSR markers, reported mean values of 0.29 (*He*) and 0.43 (*I*), which are close to those observed in the present study using polymorphic iPBS markers.

The polymorphism information content (PIC), is a metric employed for evaluating the informativeness of polymorphic loci and the discriminatory capacity of primers, considering the total number of expressed bands and their relative frequencies [36, 42]. The iPBS 2079 with the greatest PIC value (0.43) was identified as the best marker for differentiation of the present genotype. The average PIC value in this study was 0.28, which is in the middle of the reported maximum theoretical PIC, i.e., 0.5 [33]. Previous studies using different types of dominant molecular markers in *Passiflora* have reported mean PIC values of 0.24 in ISSR [17], 0.20 in AFLP [20], 0.23 in SRAP [41]. These results were lower than those obtained in our study, since most of the iPBS primers tested (58.33%) showed high PIC values (0.26 to 0.50), which clearly demonstrates that these markers have a high level of genetic diversity and potential utility in genetic studies of *Passiflora* species. In contrast [43], using RAPD and ISSR dominant markers on 20 passionfruit (*P. edulis*) genotypes, found high polymorphism information content with mean values of 0.85 to 0.88, respectively.

The cophenetic correlation coefficient obtained in our study was high, above 90%, demonstrating consistency of the fit between the graphical representation of genetic similarity and its original matrix [40]. using the dominant marker ISSR to verify the variability *in P. edulis* f. *flavicarpa* also found a high cophenetic correlation coefficient, showing a good relationship between the genetic dissimilarity and the groups formed.

Clustering analysis based on the Jaccard distance and the UPGMA method categorized the 88 *Passiflora* genotypes into ten groups. In general, a closer genetic relationship was observed between some species of the same subgenus, distributed in the same group, such as those belonging to the subgenus *Passiflora*. However, discrepancies in taxonomic classification were observed, with species of the same subgenus not grouped consistently, such as *P. suberosa* and *P. morifolia* belonging to the subgenus *Decaloba*. These results were in agreement with previous studies by other researchers who also reported discrepancies in the grouping of *Passiflora* species when using different types of markers [44]., when investigating genetic divergence in different *Passiflora* species using SSR fluorescence markers, observed that most of them were grouped with those belonging to the same subgenus, with the exception of *P. morifolia* and *P. trifasciata*, both belonging to the subgenus *Decaloba*, which was grouped with *P. edulis* (subgenus *Passiflora*). Similarly [19], when evaluating the genetic diversity of *Passiflora* using the Resistance Gene Analog (RGA) marker, found discrepancies in the taxonomic classification, with species from the same subgenus not consistently grouped together.

The iPBS-based UPGMA analysis also enabled the identification of contrasting parental genotypes that may be useful in future breeding programs aimed at developing hybrids with superior agronomic traits, such as productivity, disease resistance, fruit quality, and adaptability. The mean genetic dissimilarity among the evaluated passion fruit germplasm was 0.74, ranging from a maximum of 0.97 between *P. cincinnata* genotypes (BGP016, BGP322, and BGP413) and the commercial species *P. edulis* (BGP391), to a minimum of 0.11 between *P. edulis* genotypes (BGP224 and BGP277). The high genetic divergence observed between *P. cincinnata* and *P. edulis* highlights their potential value for broadening the genetic base of passion fruit breeding programs. These species exhibit marked differences in several morphological and physiological traits, including leaf morphology, flower coloration, fruit color at ripening, and stress-related responses, making them valuable genetic resources for selection and crop improvement. In particular, *P. cincinnata* has been reported as an important source of resistance to drought [45] and diseases affecting passion fruit crops [46]. Therefore, its incorporation into breeding programs through interspecific hybridization represents a promising strategy for developing superior genotypes with enhanced adaptability and resistance. Another important application of *Passiflora* genetic resources is the development of hybrids with ornamental potential [4]. In the present study, iPBS markers were also effective in allocating at least six ornamental hybrids to Group 9, consistent with the genetic background of their parental genotypes.

Analysis of Molecular Variance (AMOVA) was used to quantify genetic variability between and within group by clusters of *Passiflora* genotypes from the Active Germplasm Bank (BAG). The results revealed that the maximum genetic variations in passion fruit germplasm occurred within group (51%), which indicates a high degree of variation among the individuals that constitute these group, while 49% was distributed among group, suggesting that genotypes evaluated has a vast opportunity in the improvement program through a direct selection of desired traits. These findings are aligned with previous research that also reported higher genetic variations within populations in *Passiflora* [17, 40]. It is interesting to note that many factors, such as species pollination patterns, selection, gene flow between locations, and variation in genotypes, can affect genetic variation.

The results revealed significant variations (*p* < 0.01), and the majority of the variance observed occurred within group (51%), which indicates a high degree of variation among the individuals that constitute these population, while 49% was distributed among group. suggesting that genotypes evaluated has a vast opportunity in the improvement program through a direct selection of desired traits. These results are consistent with previous studies that also reported higher genetic variation within groups in *Passiflora* [47, 48].

Additionality, the AMOVA also estimated the coefficient of genetic differentiation *F*_*ST*_, which is a crucial parameter for assessing genetic variation among group and is considered a key indicator of genetic diversity. The *F*_*ST*_ (AMOVA) ranges between 0 and 0.05, which suggests negligible genetic differentiation among group; 0.05 and 0.15, which signifies a moderate degree of genetic differentiation; 0.15 and 0.25, which indicates a substantial degree of genetic differentiation; and *Fst* > 0.25, which signifies a high degree of genetic differentiation [49]. In the present study, a coefficient of genetic differentiation of *F*_*ST*_ = 0.49 (*F*_*ST*_ ˃ 0.25) was observed, indicating a high level of genetic differentiation.

The high *F*_*ST*_ value observed here (0.49) can be attributed to several biological and methodological factors. First, the *F*_*ST*_ values were calculated based on groups previously defined by clustering analysis, rather than on populations sampled from distinct geographic origins. This approach may contribute to higher *F*_*ST*_ estimates, as the groups are inherently structured according to genetic divergence, potentially leading to an overestimation of genetic differentiation. In addition, the inclusion of genotypes from different *Passiflora* species likely contributed to the high *F*_*ST*_ value observed, since interspecific divergence is expected to increase genetic differentiation. This is consistent with the low levels of admixture detected by STRUCTURE and DAPC analyses, which indicate limited gene flow among groups. Furthermore, the use of iPBS markers, which detect retrotransposon insertion polymorphisms [24], may enhance the resolution of genetic differences among genotypes, thereby contributing to elevated *F*_*ST*_ values.

It is also important to consider that iPBS markers are dominant and do not allow the direct discrimination between homozygous and heterozygous genotypes. Consequently, allele frequencies are inferred under the assumption of Hardy–Weinberg equilibrium, which may not hold in structured populations. This limitation can influence the estimation of genetic parameters, including expected heterozygosity and FST, and may introduce bias in the assessment of genetic differentiation. Therefore, the high *F*_*ST*_ observed in this study likely reflects a combination of biological factors, such as interspecific variation and restricted admixture, as well as methodological aspects related to group definition and marker characteristics.

From a biological perspective, the high *F*_*ST*_ values detected using iPBS markers have important implications for the conservation, breeding, and management of genetic resources [50]. In breeding programs, high *F*_*ST*_ values indicate substantial genetic variation that can be exploited in crosses aimed at increasing heterosis and introducing favorable alleles associated with resistance to biotic and abiotic stresses. In addition, the clear clustering pattern observed among genotypes may facilitate the planning of controlled crosses while minimizing excessive inbreeding within groups. From a conservation perspective, high *F*_*ST*_ values support the identification of genetically distinct groups that should be preserved to avoid the loss of unique alleles through genetic erosion or indiscriminate admixture. High *F*_*ST*_ values have also been reported in studies involving different plant species using retrotransposon-based markers [16, 22]. In *Passiflora edulis*, previous studies using ISSR markers reported *F*_*ST*_ values of 0.12 [40] and 0.421 [17]. Similarly, high genetic differentiation (F_ST_ > 0.25) was also reported in *Passiflora* spp. using SNP markers [20]. However, the *F*_*ST*_ values observed in the present study were higher than those previously reported.

Population structure analysis is an important technique in understanding the genetic diversity of crops [36]. Previous studies, different approaches can be employed to infer population structure in crop species in order to reveal patterns of genetic variability. In the present study, population structure was assessed using Bayesian clustering implemented in STRUCTURE and discriminant analysis of principal components (DAPC). This analysis allowed the identification of ten groups and the observed clustering pattern was consistent with the Neighbor-Joining (NJ) dendrogram. STRUCTURE analysis revealed clear genetic boundaries between populations, with minimal admixture between most groups, indicating robustness in the inferred genetic grouping.

In addition to Structure, Discriminant Analysis of Principal Components (DAPC) was employed to further investigate the genetic structure of the *Passiflora* population. This multivariate approach, which combines principal component analysis (PCA) and discriminant analysis, is effective in identifying and describing genetic differentiation among groups, making it a powerful tool for detecting subtle genetic variation among passion fruit genotypes. In the present study, DAPC successfully grouped the 88 *Passiflora* genotypes into ten clusters, showing a distribution pattern largely consistent with that inferred by Structure analysis. The clear separation among 10 groups along the first two discriminant functions (61.2%) highlights the robustness of the clustering pattern (Fig. 3c). In addition, the high assignment success rate observed in the cross-validation procedure (91%) further supports the reliability of the inferred group structure (Fig. 3b-c). Increasing the number of retained PCs beyond the optimal value resulted in a gradual decline in classification accuracy, suggesting overfitting (Fig. 3b). Moreover, both approaches revealed low levels of admixture among groups (Figs. 1e and 3c), which was also supported by the clustering pattern observed in the neighbor-joining dendrogram (Fig. 1a). This consistency among model-based, multivariate, and distance-based methods indicates a well-defined genetic structure within the evaluated germplasm and highlights the effectiveness of iPBS markers for assessing genetic diversity in *Passiflora*. Several studies have demonstrated the effectiveness of DAPC in uncovering genetic structure in different plant species [51–53], including *Passiflora* [48].

The study of genetic dispersal provided a theoretical basis for the knowledge of *Passiflora* germplasm and its use in conservation and genetic improvement strategies. Here, we sought to approach multivariate analyzes to increase the performance of markers in the analysis of genetic dispersion, even when dominant markers are used, such as iPBS-retrotransposon. The results reinforce the effectiveness of iPBS markers in revealing previously unexplored genetic diversity within the analyzed germplasm and may assist in decision-making regarding marker selection for future studies, as well as in the characterization, breeding, and management of passion fruit germplasm.

## Conclusion

This is the first study to successfully use iPBS markers to elucidate the genetic diversity and population structure of *Passiflora* spp. The number of markers analyzed was sufficient to discriminate the genotypes. Despite the dominant nature of iPBS markers, statistical analyses like DAPC, PCoA, hierarchical clustering and AMOVA provided remarkable insight into the genetic diversity among the 88 passion fruit genotypes. The efficiency of iPBS markers in detecting polymorphisms and genetic differentiation provided a solid foundation for marker-assisted breeding by enabling the identification of genetically contrasting genotypes, such as *P. cincinnata* accessions (BGP016, BGP322, and BGP413) and *P. edulis* (BGP391), which exhibited a high genetic dissimilarity value (0.97). This finding suggests that these genotypes may represent promising parental candidates for future breeding programs aimed at developing productive passion fruit hybrids with enhanced resistance to abiotic stresses. Future studies may further explore the *Passiflora* Active Germplasm Bank (BAG) through the integrated analysis of molecular data based on iPBS retrotransposon markers and phenotypic traits, thereby improving strategies for the conservation and use of available genetic resources.

## Supplementary Information

Below is the link to the electronic supplementary material.

Supplementary Material 1

Supplementary Material 2

## Data Availability

The datasets generated or analyzed in the current study are available from the corresponding author upon reasonable request.

## Abbreviations

iPBS: inter-primer binding site
DAPC: Discriminant analysis of principal components
PIC: Polymorphic information content
TNB: Total number of bands
GNNA: Genotype numbers that did not amplify
NSB: Number shared bands by more than one genotype
*He*: Nei’s genetic diversity
*I*: Shannon’s information index
