# Supplementary figures and images for: Genetic diversity and population structure of Passiflora spp. using inter-primer binding site (iPBS) – retrotransposon markers
Source: Mol Biol Rep. 2026 Jun 1;53(1):867. doi: 10.1007/s11033-026-12038-9 (PMC13226455; doi:10.1007/s11033-026-12038-9)

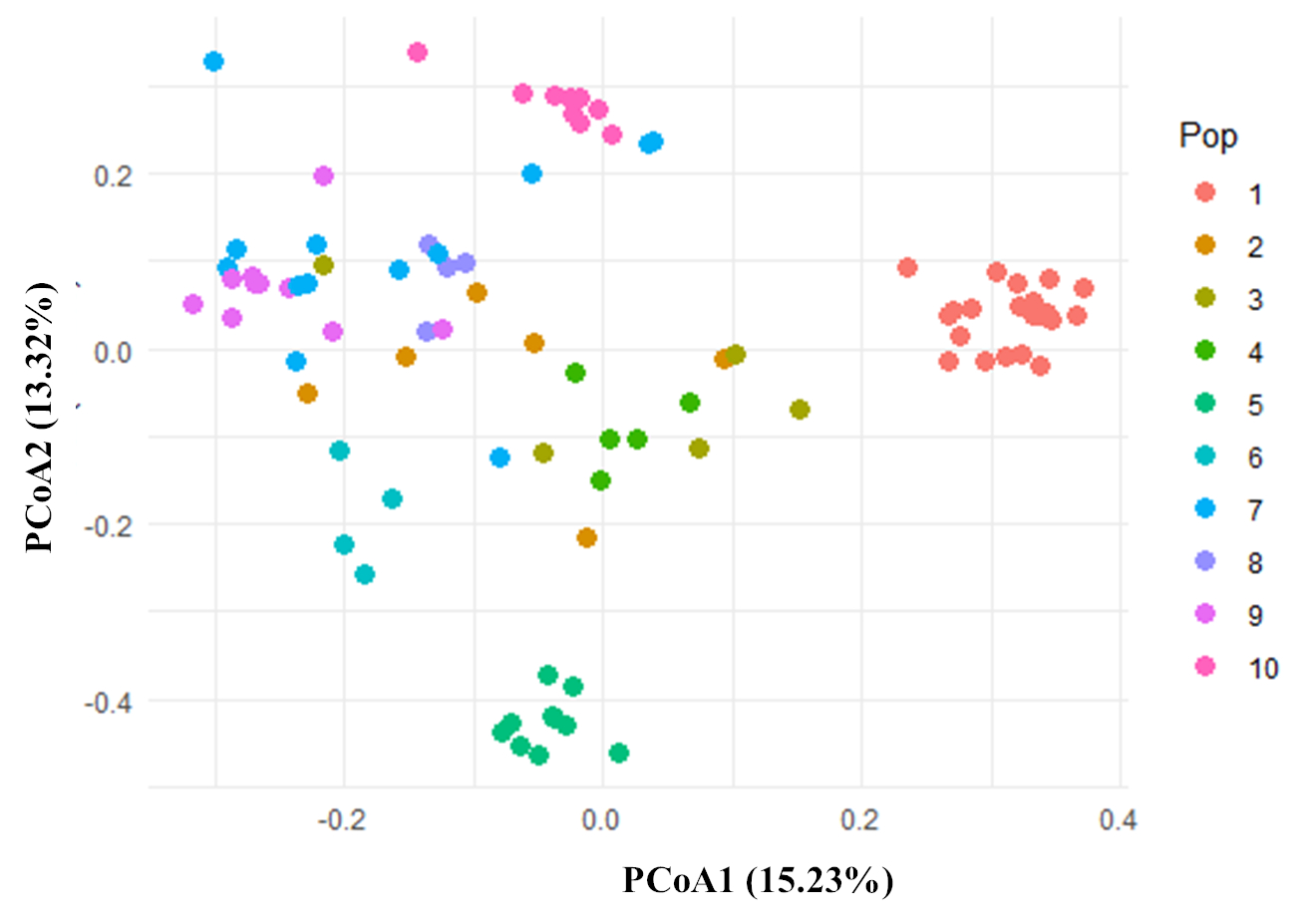

Supplement: Supplementary file 1 — Supplementary Material 1 [file 11033_2026_12038_MOESM1_ESM.jpeg]
